# Detection and Localization of Excavation-Disturbance-Related Near-Field Microseismic Events During TBM Tunneling

**DOI:** 10.3390/s26134163

**Published:** 2026-07-02

**Authors:** Jiawei Song, Qi Li, Chenyang Zhu, Yue Zhang, Guowei Zhu

**Affiliations:** 1 College of Geoscience and Surveying Engineering, China University of Mining and Technology-Beijing, Beijing 100083, China; songjiaweiiiii@163.com (J.S.); 17866562865@163.com (Q.L.); zy69177933@163.com (Y.Z.); zgw@cumtb.edu.cn (G.Z.); 2 State Key Laboratory of Water Resource Protection and Utilization in Coal Mining, National Institute of Low Carbon and Clean Energy, Beijing 102209, China; 3 State Key Laboratory of Coal Fine Exploration and Intelligent Development, China University of Mining and Technology-Beijing, Beijing 100083, China

**Keywords:** terrestrial continuous sensing, TBM-noise-based seismic detection, three-component near-field array, structural activation, advance geological prospecting

## Abstract

Tunnel boring machine (TBM) excavation generates continuous mechanical vibration that can obscure weak, short-duration propagating responses related to structural-plane disturbance. This study develops a Signal-Constrained Activation Detection and Localization (SCADL) framework using continuous three-component geophone-array records. SCADL first constructs an adaptive multi-station consistency trigger from synthesized three-component envelopes and rejects non-propagating mechanical disturbances using coherence and polarization constraints. First arrivals are picked by fusing statistical abrupt-change, local onset-gradient, and polarization-variation evidence, and event locations are estimated using an ahead-of-face layered velocity model and relative correction of similar event pairs. A multi-evidence activation index then integrates spatial clustering, coupling with the face-disturbance zone, shear/compression energy ratio, temporal evolution, and event quality to identify high-confidence candidate structural-plane activation events. The workflow was evaluated using one 16 h continuous field monitoring record acquired from a single TBM monitoring section and manually reviewed reference sets comprising 286 propagating events, 136 high-confidence events for arrival-time evaluation, and 96 events for activation-assessment review. SCADL identified 263 valid propagating events, achieved an event-level F1-score of 0.88, reduced the median arrival-time picking error to 2.4 ms, constrained the localization residual to 2.9 ms, and compressed the corrected cluster thickness to 0.82 m. Among the detected events, 86 high-confidence candidate activation events formed two clusters spatially consistent with the F04 and F02 structural zones confirmed by post-excavation geological validation. These results support the feasibility of SCADL for single-section TBM monitoring.

## 1. Introduction

Terrestrial continuous sensing for underground engineering is expanding from geometric deformation observation to near-field dynamic response analysis [1,2,3,4,5,6,7]. Unlike remote-sensing observations that target surface displacement or deformation, three-component geophone arrays in TBM excavation directly record continuous vibration responses of the surrounding rock around underground tunnels. They can therefore recover event propagation characteristics, spatial positions, and coupling relationships with structural planes from continuous signals [8,9,10,11,12,13]. For identifying ahead-of-face anomalous zones and evaluating surrounding-rock stability, the key question is whether propagating events associated with disturbance-induced structural activation can be separated robustly from a strong mechanical background and whether interpretable spatial localization results can be obtained.

Existing studies have proceeded along two lines: engineering microseismic interpretation and terrestrial monitoring methods. On the one hand, TBM microseismic studies indicate that event spatiotemporal clustering, b-value, energy index, and relative correction of similar event pairs are informative indicators of surrounding-rock stability and rockburst development [14,15,16,17,18,19,20,21]; existing cases in deep-buried tunnels show that instability is often accompanied by enhanced event clustering, elevated energy release, and a decrease in the b-value. On the other hand, within the broader field of civil-engineering remote sensing, ground-based radar, laser scanning, and near-field sensor arrays share the objective of converting continuous sensing data into verifiable structural information [22,23]. In contrast, TBM scenarios still lack a unified signal-interpretation workflow that starts from continuous three-component sensing records and simultaneously completes event detection, localization, and structural activation recognition [24,25,26,27,28,29,30].

For vibration monitoring systems under TBM excavation conditions, the raw input is often a multi-hour continuous three-component record that simultaneously contains cutterhead vibration, mechanical impacts, and environmental noise [31]. Target events are not only weak, but in many cases exhibit interpretable propagation relationships only across a subset of adjacent stations. Directly applying a fixed threshold or single-station triggering method often produces many false alarms [32,33]. If subsequent statistics are conducted without propagation-consistency screening, mechanical noise can easily enter the event catalog, weakening the credibility of localization and risk interpretation.

To address these issues, a vibration-signal interpretation method is needed for underground engineering under TBM excavation conditions. Its objective consists of two levels. The first is the sensing-detection level: potential valid propagating events are screened from continuous three-component records, while equipment noise and mechanical-impact interference are removed as far as possible. The second is the velocity-model-constrained spatial interpretation level: the layered velocity model obtained from advance geological prediction is used to obtain constrained spatial interpretation results for event clusters.

The core idea of this study is to identify propagating events that may be generated by structural-plane activation within the TBM-disturbance background. The workflow can be divided into the following parts:

(1) For continuous three-component terrestrial sensing records, an adaptive multi-station consistency trigger and coherence-polarization joint screening workflow are proposed. Candidate events are defined by propagation consistency rather than by amplitude anomalies alone, thereby improving the detection reliability of near-field propagating events under a strong mechanical background.
(2) A first-arrival picking strategy jointly constrained by statistical abrupt change, local onset gradient, and three-component polarization abrupt change is proposed. The propagation order of adjacent stations is used to check the consistency of the picking results, providing stable station-level arrival constraints for subsequent localization.
(3) Under the layered velocity model observed by advance geological prospecting, a spatial interpretation method combining arrival-time residual search with relative correction of similar event pairs is constructed to obtain stable event clusters.
(4) A candidate structural activation index is constructed by integrating spatial clustering, face-disturbance coupling, and temporal statistical evolution.

## 2. Materials and Methods

### 2.1. Engineering Background and Observation System

The field record used in this case study consists of 16 h of continuous three-component geophone data sampled at 1 kHz. The inputs used by the workflow are the continuous waveforms, station coordinates, sampling parameters, monitoring time, and an ahead-of-face layered velocity model constrained by advance prospecting, borehole information, and rock-specimen velocity tests.

Because the 12 stations are deployed at 1 m spacing, the main text uses a compact face-local coordinate system rather than listing all absolute coordinates. The tunnel face at 5000 m is taken as the origin; the X-axis is positive ahead of the face, and the Y- and Z-axes denote lateral and vertical offsets.

Under this limited-aperture geometry, localization is constrained by station coordinates, first-arrival times, cross-correlation time shifts, and the ahead-of-face velocity model. The processing order is as follows: the layered velocity model is first established from advance prospecting, borehole information, core logging, and rock-specimen velocity tests; continuous waveforms are then screened and picked; a fixed station static correction may be applied to compensate for repeatable timing offsets; and the corrected arrivals are finally used for velocity-model-constrained location search and relative correction of similar event pairs. The post-excavation geological sketch is reserved only for posterior validation of event clusters.

The 2.9 ms residual reported in this study is therefore a corrected localization residual obtained after the fixed within-record timing correction, not an independent uncorrected residual and not a standalone proof of absolute velocity-model accuracy. Because the present single-session revision materials do not contain a separate calibration/evaluation split or an uncorrected residual rerun, possible circularity cannot be fully excluded.

### 2.2. Data Representation and Face-Local Coordinate System

The raw input is a continuous three-component vibration sequence rather than a presegmented event catalog. Let the three-component record of the i-th station at time t form the vector $u_{i}(t)=(u_{ix}(t),u_{iy}(t),u_{iz}(t){)}^{⊤}$. Within a sliding time window of length $T_{w}$, the continuous record can be expressed explicitly in tensor form as follows:

$$X^{\left(w\right)}\in R^{N\times C\times T},X_{i,c,j}^{\left(w\right)}=u_{ic}(t_{j}),i=1,\ldots ,N,c=1,\ldots ,C,j=1,\ldots ,T.$$

where N=12 is the number of stations, C=3 is the number of components, $T=T_{w}f_{s}$ is the number of samples per window, $t_{j}=j/f_{s}$ is the discrete sampling time, and $f_{s}=1000\;Hz$ is the sampling frequency. This study organizes the continuous records using a 0.5 s sliding window and a 0.05 s sliding step, so that the detection module can cover short-duration impulsive events while preserving interstation propagation relationships.

Station indices are denoted by i, sample/time indices by j and τ, event indices by k and l, and statistical-window indices by w. Event-level quantities use the event subscript, window-level quantities use the window subscript, vectors are treated consistently as three-component records or spatial coordinates, and normalized variables are defined at their first use and then used with the same subscript convention throughout Section 2.

**Figure 1:**
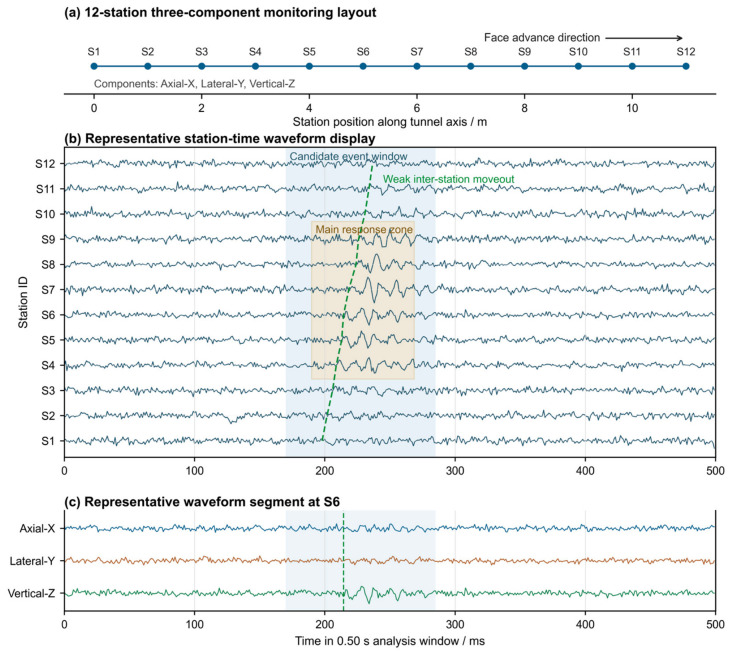
Simplified representation of the continuous three-component input data. (**a**) Linear 12-station monitoring layout and the three recorded components at each station; (**b**) station–time waveform display of a representative candidate window after three-component amplitude synthesis. Each trace corresponds to one station, the horizontal axis shows a 0.50 s analysis window, the blue band marks the main event-response interval, and the dashed line indicates the across-station arrival sequence. (**c**) Representative waveform segment at S6.

To make the input data structure easier to interpret, Figure 1 shows the continuous records as a station–time waveform display rather than as a three-dimensional tensor rendering. The monitoring input consists of 12 stations, each recording axial, lateral, and vertical components, and the detection module scans the continuous stream using overlapping 0.50 s windows. In Figure 1, each row represents one station, the horizontal axis denotes time within the analysis window, the blue band marks the main event-response interval, and the dashed trajectory indicates the relative arrival order of a weak propagating event across adjacent stations.

Event localization and interpretation use the face-local coordinate system. Let the position of the tunnel face in the engineering coordinate system be $x_{f}$, and let the engineering coordinates of an event source be x. Its local coordinates are then defined as $\xi =x-x_{f}$. Here, $\xi_{x}>0$ indicates that the event is located ahead of the tunnel face, whereas $\xi_{x}<0$ indicates that the event is behind the face; $\xi_{y}$ and $\xi_{z}$ correspond to lateral and vertical offsets, respectively. The localization plots and case tables in the main text all use ξ to express event locations, highlighting the geometric relationship with the target body ahead of the face.

### 2.3. Adaptive Multi-Station Consistency Trigger

Candidate windows are first extracted from the continuous records by combining three-component amplitude synthesis with a multi-station consistency trigger. This step is designed to retain weak propagating responses while suppressing isolated mechanical spikes and single-station polarity effects.

(1) $$A_{i}(t)=\sqrt[{2}]{u_{ix}^{2}(t)+u_{iy}^{2}(t)+u_{iz}^{2}(t)}$$

For the i-th station at time t, the three-component synthesized amplitude $A_{i}(t)$ is defined by Equation (1). Here, $u_{ix}(t)$, $u_{iy}(t)$, and $u_{iz}(t)$ are the axial, lateral, and vertical component records, respectively. This synthesis makes the subsequent statistics independent of single-component polarity.

**Figure 2:**
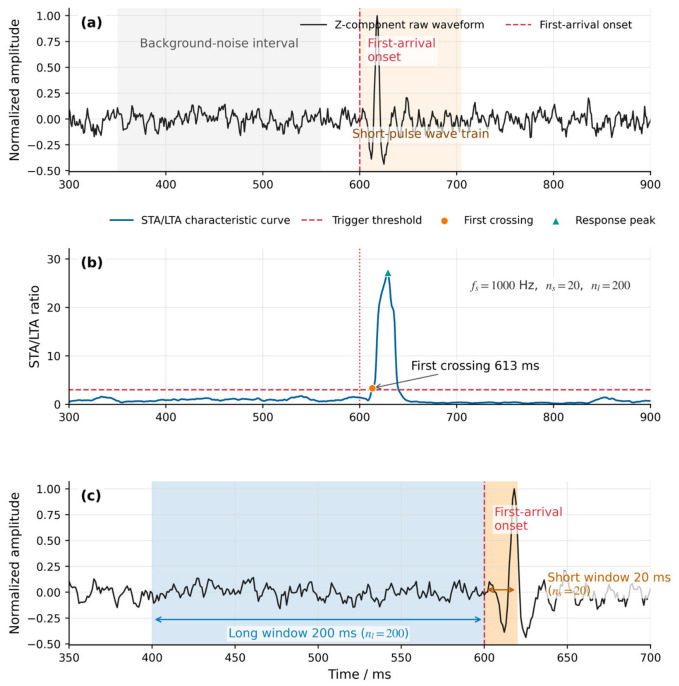
Representative short-duration propagating-event waveform and its STA/LTA response at a 1 kHz sampling rate, with n_s = 20 and n_l = 200. (**a**) Raw waveform and first-arrival onset; (**b**) STA/LTA trigger response and threshold crossing; and (**c**) short- and long-window configuration for onset detection.

A conventional STA/LTA-type ratio is then computed from the synthesized amplitude using 20 and 200 samples, with a small denominator-stabilizing constant. Because this operation is a standard triggering procedure, its separate STA, LTA, and ratio equations have been omitted. The representative waveform and STA/LTA parameter configuration are shown in Figure 2.

(2) $$Q(t)=\alpha {max}_{1\leq i\leq N}R_{i}(t)+(1-\alpha )\frac{1}{K}\sum_{i\in \Omega_{K}(t)}R_{i}(t)$$

Equation (2) defines the joint trigger score Q(t). Here, N=12 is the number of stations, and $\Omega_{K}(t)$ denotes, at time t, the set of the top K stations with the largest trigger values; this study sets K=4. The coefficient α is a balancing parameter, and this study sets α=0.55. The first term retains the principal response that is most sensitive to weak events, whereas the second term emphasizes the common uplift of adjacent stations.

(3) $$\Gamma (t)=Q_{0.75}^{\left(W\right)}(t)+\kappa [Q_{0.75}^{\left(W\right)}(t)-Q_{0.25}^{\left(W\right)}(t)]$$

Equation (3) gives the time-varying trigger threshold Γ(t). Here, $Q_{0.75}^{\left(W\right)}(t)$ and $Q_{0.25}^{\left(W\right)}(t)$ are the upper and lower quartiles, respectively, of the joint score in the background window centered at t with length W=120 s; κ is the threshold amplification coefficient, and this study sets κ=1.8. Therefore, Γ(t) adapts to the background-noise level.

(4) $$S_{e}=\frac{[\sum_{i=1}^{N_{e}}{\underset{A}{\sim }}_{i}(t+\tau_{i}){]}^{2}}{N_{e}\sum_{i=1}^{N_{e}}{\underset{A}{\sim }}_{i}^{2}(t+\tau_{i})}$$

Equation (4) defines the event-level coherence score. Station-alignment shifts are obtained using standard normalized cross-correlation within the candidate window (±2 ms in this study); therefore, the cross-correlation formula is not displayed separately.

When the joint trigger score exceeds the time-varying threshold, this study extracts a 0.50 s time window as the candidate event segment, extending 0.12 s before and 0.38 s after the trigger point. Propagation consistency is then calculated using Equation (4).

**Figure 3:**
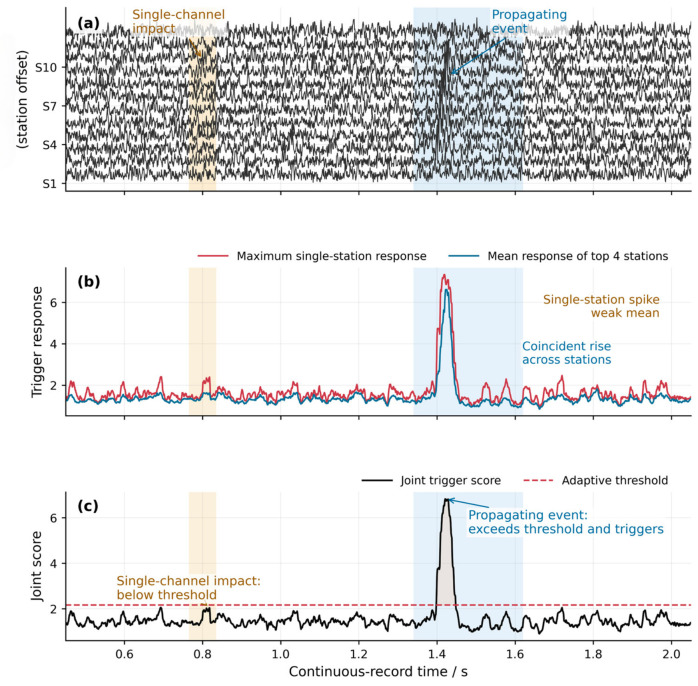
Processing effect of adaptive multi-station consistency triggering. (**a**) Continuous multi-station waveform record with a single-channel impact and a propagating event; (**b**) comparison between the maximum single-station response and the mean response of the top four stations; and (**c**) joint trigger score with the adaptive threshold.

Figure 3 summarizes the role of the adaptive multi-station trigger: isolated spikes may raise a single-station response, whereas retained candidates must show simultaneous multi-station uplift and propagation coherence.

### 2.4. Coherence-Polarization Screening of Candidate Events

After triggering, coherence and polarization screening are applied to reject high-amplitude mechanical disturbances. Coherence tests the interstation propagation relationship, and polarization checks whether the initial motion is consistent with a near-field P-wave response.

$$T_{p}=L_{p}\Delta t=\frac{L_{p}}{f_{s}}$$

This study uses $L_{p}=20$ samples; therefore, under $f_{s}=1000\;Hz$, $T_{p}=20/1000=20\;ms$. This time scale can cover the polarization abrupt-change process before and after the first arrival while avoiding mixing effects from later wave segments caused by an overly long window.

(5) $${\underset{P}{¯}}_{e}=\frac{1}{M_{e}}\sum_{i\in \Omega_{e}}{max}_{t\in W_{e}}P_{i}(t)$$

Equation (5) defines the event-level mean peak polarization index used for screening. The station-level covariance and eigenvalue calculations follow conventional three-component polarization analysis [10,11,12].

**Figure 4:**
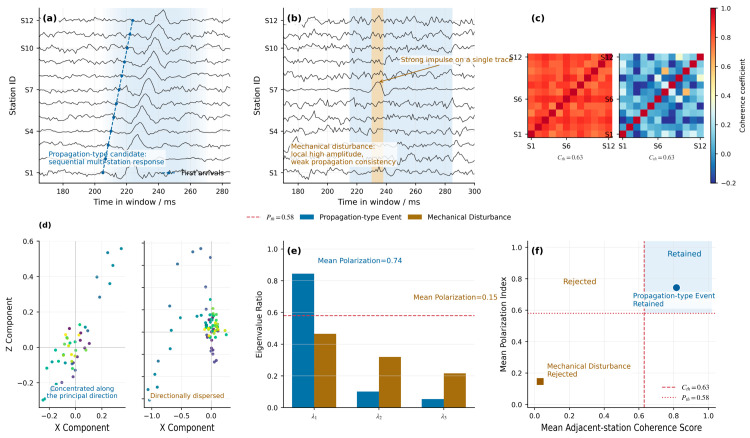
Effect of coherence-polarization screening for candidate events. The propagating candidate event shows an across-station arrival-time order and more concentrated covariance eigenvalues, whereas the mechanical-disturbance window is dominated by a strong single-trace impulse and weak propagation consistency. (**a**) Propagation-type candidate waveform; (**b**) mechanical-disturbance waveform; (**c**) interstation coherence matrix, where color intensity represents the normalized correlation coefficient; (**d**) three-component particle-motion projection, where colored circles represent station-level particle-motion samples; (**e**) covariance-matrix eigenvalue ratios; and (**f**) joint coherence-polarization screening, where blue points indicate retained propagating candidates and orange points indicate rejected mechanical disturbances.

Equations (4) and (5) therefore convert amplitude-triggered candidates into propagation-consistent candidates. Figure 4 contrasts a retained propagating window with a rejected mechanical-disturbance window under the same coherence-polarization criteria.

### 2.5. Joint First-Arrival Picking Using Onset-Gradient Abrupt Change and Polarization

For each retained candidate, first arrivals are picked by combining statistical change, onset gradient, and polarization change in a single score. The standard piecewise-variance, local-gradient, and min-max normalization formulas are not displayed separately; only the fused picking score and final pick rule are retained.

(6) $$J_{i}(\tau )=\left[1-K_{i}^{n}(\tau )\right]+\beta_{1}P_{i}^{n}(\tau )+\beta_{2}G_{i}^{n}(\tau )$$

Equation (6) combines the normalized statistical-change, polarization, and gradient evidence into one picking score. The polarization and gradient weights are set to 0.35 and 0.20, respectively, so that the accepted pick corresponds to the sample where the onset-related evidence terms coincide.

(7) $$\tau_{i}^{*}={argmax}_{\tau \in [1,L]}J_{i}(\tau ),t_{i}^{obs}=t_{w,0}+\tau_{i}^{*}\Delta t$$

Equation (7) gives the final first-arrival time for each station. Stations whose picking-time differences are inconsistent with the cross-correlation shifts are marked as low confidence and assigned lower weights during localization.

This picking module is rule-based rather than label-driven; it uses onset statistics and polarization consistency to stabilize picks for weak short-pulse events.

**Figure 5:**
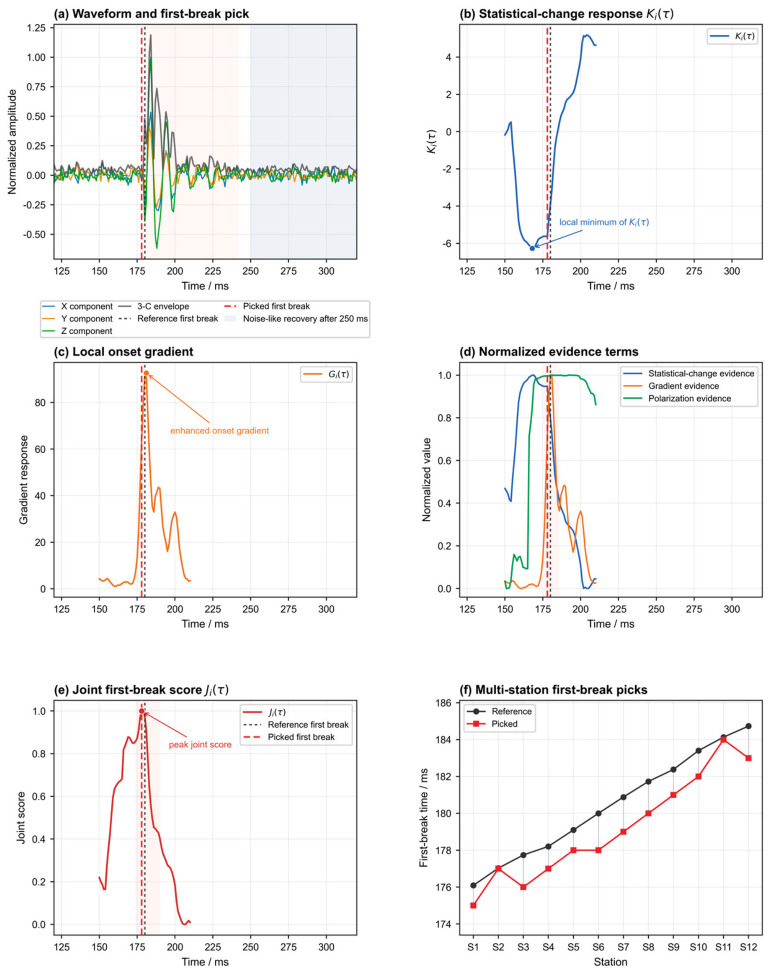
First-arrival picking process jointly constrained by statistical abrupt change, onset gradient, and polarization. (**a**) Representative three-component waveform and picked first arrival, where the light red background marks the onset-related response interval and the light blue background marks the noise-like recovery interval; (**b**) piecewise statistical-change response; (**c**) local onset-gradient response; (**d**) normalized statistical-change, gradient, and polarization evidence; (**e**) fused joint picking score; and (**f**) multi-station first-arrival picking results.

Figure 5 illustrates how statistical abrupt change, onset gradient, and polarization jointly constrain first-arrival picking. Figure 5a shows that the representative event has a clear onset near 180 ms, then forms a short-duration impulsive response and largely decays to the background noise level before 250 ms. This indicates a short-duration waveform characteristic commonly seen in near-field weak events rather than a long mechanical oscillation. In Figure 5b, the piecewise statistic $K_{i}(\tau )$ has a local minimum near the first arrival, indicating that when the candidate split point approaches the true first-arrival onset, the statistical difference between the two waveform segments before and after the split is most pronounced. Figure 5c further shows that the local onset gradient increases rapidly after the first arrival and can characterize the short-time transition from background noise to an effective impulsive response.

On this basis, Figure 5d normalizes the piecewise statistic, local gradient, and polarization feature so that the three types of evidence are comparable. The statistical abrupt-change term mainly constrains the overall change before and after the first arrival, the gradient term is more sensitive to amplitude growth at the onset instant, and the polarization term reflects the concentration of three-component particle-motion direction near the first arrival. After the three terms are fused by Equation (6), the joint score $J_{i}(\tau )$ forms a distinct peak near the first-arrival onset, as shown in Figure 5e. Finally, the multi-station picking results maintain a continuous propagation order in space and are generally consistent with the reference arrival times, indicating that the method can jointly use statistical abrupt change, local onset, and polarization-concentration information in short-pulse weak events to improve the stability and interpretability of first-arrival picking.

**Figure 6:**
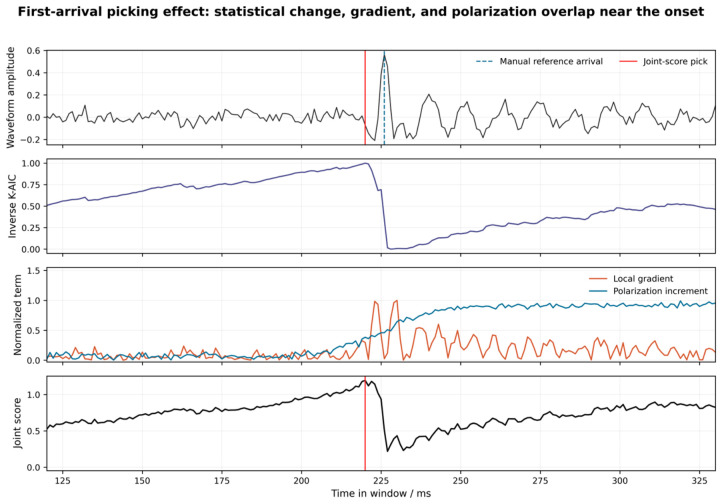
Processing effect of joint first-arrival picking based on statistical abrupt change, onset gradient, and polarization. (**a**) Waveform segment with the manual reference arrival and joint-score pick; (**b**) inverse K-AIC response; (**c**) normalized local-gradient and polarization-increment terms; and (**d**) fused joint picking score. In (**b**,**d**), the vertical reference/pick lines mark the arrival-time constraints used to compare the individual evidence terms and the fused score.

Figure 6 provides a compact visual check of the picking score and its three evidence terms near the selected first-arrival time.

### 2.6. Advance Velocity-Model-Constrained Localization and Relative Correction of Similar Event Pairs

Because the array is short and nearly linear, the localization results are interpreted primarily as velocity-model-constrained event clusters rather than unconstrained high-precision three-dimensional hypocenters. The procedure combines layered-model travel times, arrival residual search, and relative correction of similar event pairs.

The resulting locations are therefore evaluated at the event-cluster scale. The axial position is mainly constrained by interstation arrival order and the ahead-of-face travel-time gradient, whereas the lateral and vertical coordinates have lower resolution because the stations are distributed almost linearly. Localization uncertainty is assessed using four diagnostics: the station-level first-arrival picking error, the arrival-time residual after velocity-model-constrained search, the cluster thickness after relative correction of similar event pairs, and the mean location drift obtained by station-removal testing. These diagnostics are used to interpret the locations as constrained clusters rather than precise independent point hypocenters; the corresponding quantitative results are reported in the Results section after the detection metrics.

(8) $$V=\left\{(\Omega_{l},v_{l})|l=1,\ldots ,L\right\},v(s)=v_{l},\;s\in \Omega_{l}$$

Equation (8) defines the layered velocity model used for localization. Here, $\Omega_{l}$ is the l-th velocity zone, $v_{l}$ is the P-wave velocity of that zone, L is the number of zones, and s is the spatial position vector. This model is jointly established from advance geological prospecting/prediction, advance boreholes, core logging, and wave-velocity tests on rock specimens, and can be obtained before or during excavation.

The layered velocity model is therefore used as an a priori travel-time approximation rather than as an exact three-dimensional structural surface. If the true geological boundaries ahead of the face are curved, dipping, discontinuous, or oblique to the tunnel axis, the calculated travel-time field will contain geometry-related bias. Such bias may be partly absorbed by the fixed within-record timing correction and by relative correction of similar event pairs, but it can still increase the corrected arrival-time residual and shift the final event clusters along the tunnel-axis or weakly constrained lateral and vertical directions.

(9) $$\Psi =\sum_{\left(p,q\right)}\sum_{i=1}^{N}\omega_{i,pq}(\Delta t_{i,pq}^{obs}-\Delta t_{i,pq}^{cal}{)}^{2}$$

Equation (9) gives the relative correction objective for similar event pairs. The observed and calculated event-pair time differences are formed from station-level first arrivals and layered-model travel times, respectively; the reliability weight depends on waveform similarity and picking consistency.

The travel-time table is built over 0–40 m ahead of the face, +/−2.5 m laterally, and +/−2.0 m vertically. The search range defines the interpretation volume and does not prescribe the positions of F04 or F02.

**Figure 7:**
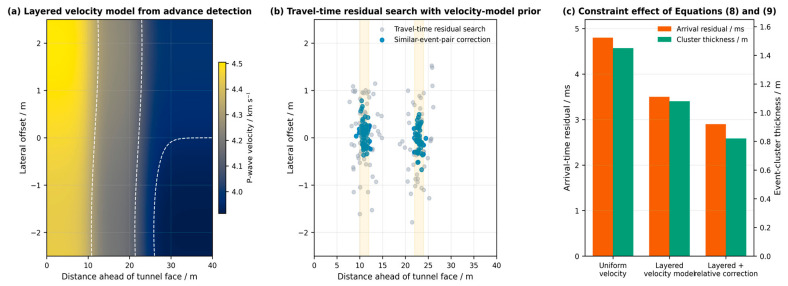
Processing effect of advance velocity-model-constrained localization and relative correction of similar event pairs. (**a**) Layered P-wave velocity model used as the travel-time prior; (**b**) event-position distribution from travel-time residual search and after similar-event-pair correction; and (**c**) reduction in arrival-time residual and event-cluster thickness under the sequential constraints of Equations (8) and (9).

Figure 7 illustrates how the layered velocity prior and similar-event correction reduce the spatial dispersion that would otherwise arise from velocity-position coupling in the short-aperture array.

### 2.7. Multi-Evidence Structural Activation Recognition

Candidate structural activation is identified with a multi-evidence index rather than a single amplitude or location threshold. The index integrates spatial clustering, face-disturbance coupling, mechanism information, statistical evolution, and event quality. Because the mechanism and temporal-evolution terms use amplitude-derived quantities, the relative magnitude, shear-to-compression energy ratio, cumulative apparent-volume proxy, and completeness magnitude are defined explicitly below. The b-value decrease is then calculated using the Gutenberg−Richter maximum-likelihood approach only as a normalized temporal-evolution indicator.

Because the 12-station near-field array was not documented as absolutely amplitude-calibrated, all amplitude-derived quantities are used as internally normalized relative indicators. They are used to compare events within this 16 h record and should not be interpreted as calibrated seismic magnitude, radiated energy, or absolute source volume.

For event k, the synthesized three-component amplitude at station i is first obtained from the vector norm of the recorded components. The distance-normalized event envelope and the relative magnitude are then defined by Equation (10):

(10) $$a_{i}(t)={\left\|u_{i}(t)\right\|}_{2},A_{k}={median}_{i\in S_{k}}\{r_{ik}{max}_{t\in W_{k}}a_{i}(t)\},M_{k}={log}_{10}(\frac{A_{k}+\varepsilon }{A_{ref}+\varepsilon })$$

Here, $S_{k}$ is the set of valid stations for event k, $r_{ik}$ is the source-station distance from the velocity-model-constrained location, $W_{k}$ is the event window, $A_{ref}$ is the median distance-normalized envelope of the accepted propagating-event catalog, and ε prevents singular values. Thus, $M_{k}$ is a relative magnitude used only for within-record frequency-magnitude statistics.

The shear-to-compression energy ratio is computed from squared three-component amplitudes in fixed P-dominated and later S/coda-dominated windows around the picked onset using Equation (11):

(11) $${\left(E_{s}/E_{p}\right)}_{k}=\frac{\Sigma_{i\in S_{k}}\int_{W_{S,k}}a_{i}^{2}(t)dt}{\Sigma_{i\in S_{k}}\int_{W_{P,k}}a_{i}^{2}(t)dt+\varepsilon },E_{k}^{*}=\Sigma_{i\in S_{k}}\int_{W_{k}}a_{i}^{2}(t)dt$$

The resulting ratio is a mechanism-sensitive relative feature. It reduces the dependence on absolute gain but remains affected by sensor coupling, window selection, and near-field radiation; therefore, it is used as one evidence term rather than as an independent focal-mechanism solution.

The apparent-volume term is treated as a normalized proxy derived from the relative magnitude, and the cumulative apparent-volume proxy in statistical window w is calculated by summing the event-level proxies using Equation (12):

(12) $$V_{A,k}^{*}=10^{1.5(M_{k}-M_{ref})},{CAV}_{w}^{*}=\Sigma_{k\in E_{w}}V_{A,k}^{*}$$

The superscript * denotes a relative proxy. Without instrument response correction and absolute amplitude calibration, $V_{A,k}^{*}$ and ${CAV}_{w}^{*}$ are not reported as absolute apparent volume; they only describe relative changes in the same monitoring session.

For the Gutenberg−Richter fit, the completeness magnitude is selected from the maximum-curvature point and the stable linear part of the cumulative frequency-magnitude curve of the retained catalog. With a bin width of 0.10 relative-magnitude units, the non-cumulative distribution reaches its stable high-frequency branch near −0.50 and the cumulative log-frequency relation is approximately linear above this value. Therefore, the same $M_{min}$ is held fixed for all statistical windows, and the window-scale b-value is calculated by Equation (13):

(13) $$M_{min}=-0.50,\Delta M=0.10,b_{w}=\frac{{log}_{10}(e)}{mean(M_{k}|M_{k}\geq M_{min})-(M_{min}-\frac{\Delta M}{2})}$$

Keeping $M_{min}$ fixed avoids retuning the Gutenberg−Richter fit in each time window. If calibrated amplitudes become available, the relative magnitude, S/P energy ratio, and apparent-volume proxy should be replaced by calibrated source-parameter estimates.

(14) $$D_{k}=\sum_{l\neq k}exp\left[-\frac{{\left\|\xi_{k}-\xi_{l}\right\|}^{2}}{2h^{2}}\right],M_{c,k}=\frac{D_{k}-D_{min}}{D_{max}-D_{min}+\varepsilon },M_{f,k}=exp\left(-\frac{d_{f,k}}{l_{f}}\right)$$

Equation (14) defines the spatial clustering term $M_{c,k}$ and the face-disturbance coupling term $M_{f,k}$. Here, $D_{k}$ is the kernel density of the k-th event in the relative localization space; $\xi_{k}$ and $\xi_{l}$ are the local coordinates of events k and l, respectively; h is the spatial kernel width; $d_{f,k}$ is the shortest distance from the event to the disturbance-affected zone $\Omega_{f}$ ahead of the tunnel face; $l_{f}$ is the distance-decay scale; and $M_{c,k}$ is derived from the event cluster itself.

(15) $$M_{m,k}=clip(\frac{{log}_{10}(E_{s}/E_{p}{)}_{k}-{log}_{10}\eta_{1}}{{log}_{10}\eta_{2}-{log}_{10}\eta_{1}},0,1)$$

In Equation (15), $E_{s}/E_{p}$ is the shear-to-compression energy ratio of the k-th event, and $\eta_{1}$ and $\eta_{2}$ are the two mechanism-boundary reference values used consistently in the equation denominator and in the following definition. According to studies on tunnel microseismic mechanism recognition and rockburst monitoring, smaller $E_{s}/E_{p}$ values usually correspond to tensile-dominated failure, intermediate values to mixed failure, and higher values to shear-dominated failure.

(16) $$M_{e,w}=\nu_{1}{\underset{N}{\sim }}_{w}+\nu_{2}{\underset{EI}{\sim }}_{w}+\nu_{3}{\underset{CAV}{\sim }}_{w}+\nu_{4}{\underset{B}{\sim }}_{w}$$

Equation (16) defines the statistical-evolution term M_e,w. Here, the window event count, relative energy index, cumulative apparent-volume proxy, and b-value decrease are first normalized by interval scaling and then combined using weights of 0.25, 0.25, 0.20, and 0.30, respectively. The relative energy index is defined from the median event energy in the window and the background segment, and the cumulative apparent-volume proxy is the sum of event-level apparent-volume proxies in the same window.

(17) $$M_{q,k}=0.5exp(-\frac{{\underset{e}{¯}}_{t,k}}{e_{0}})+0.5exp(-\frac{r_{loc,k}}{r_{0}})$$

Equation (17) defines the event-quality term $M_{q,k}$. Here, ${\underset{e}{¯}}_{t,k}$ is the mean first-arrival uncertainty of all valid stations for the k-th event, $r_{loc,k}$ is its localization residual, and $e_{0}$ and $r_{0}$ are scale parameters. This study sets $e_{0}=5\;ms$ and $r_{0}=5\;ms$.

(18) $$I_{sa,k}=M_{q,k}\left(\omega_{1}M_{c,k}+\omega_{2}M_{f,k}+\omega_{3}M_{m,k}+\omega_{4}M_{e,w(k)}\right)$$

Equation (18) gives the candidate structural activation index $I_{sa,k}$ of the k-th event. Here, $M_{e,w(k)}$ denotes the evolution term of the statistical window to which event k belongs, and $\omega_{1}$ to $\omega_{4}$ are the weights of spatial clustering, disturbance coupling, mechanism term, and statistical-evolution term, respectively. This study sets $\omega_{1}$ = 0.32, $\omega_{2}$ = 0.18, $\omega_{3}$ = 0.24, and $\omega_{4}$ = 0.26. Finally, τ1=0.58 and τ2=0.76 are used as classification thresholds. The key parameter values used in this section are listed in Table 1.

**Figure 8:**
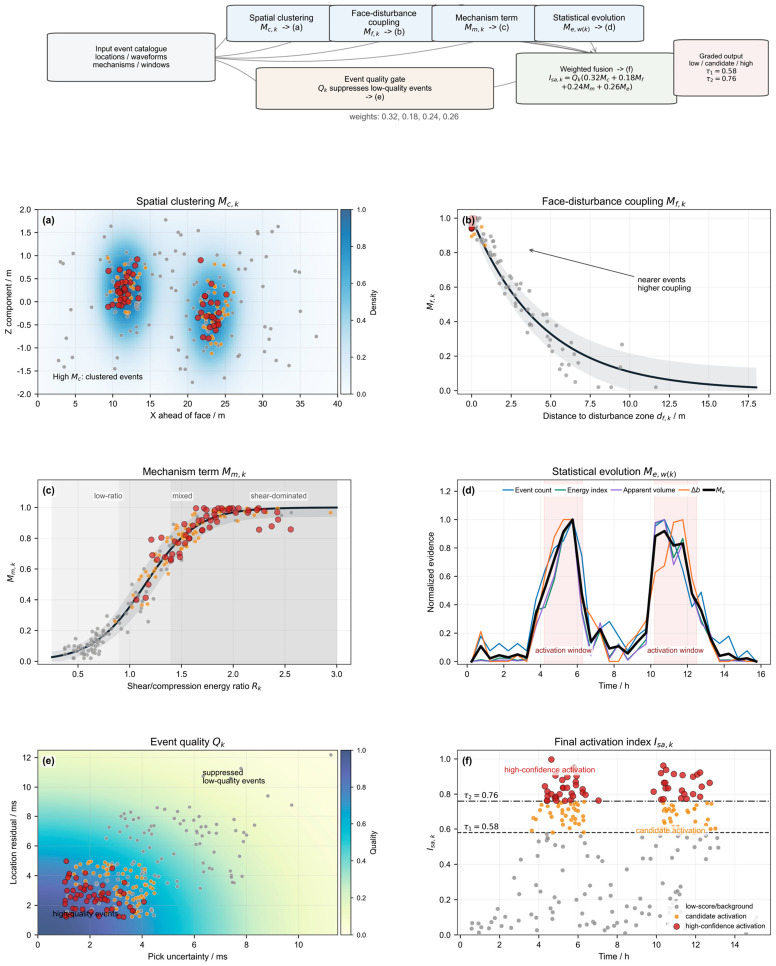
Mechanism-evidence coupling framework for multi-evidence candidate structural activation recognition. The upper part shows the evidence-convergence mechanism of the candidate structural activation index, and the lower part shows the response of each evidence term: (**a**) event spatial distribution and kernel-density response, where red/orange balls denote high-confidence/candidate activation events and gray balls denote low-index background events; (**b**) the face-disturbance coupling term decays as the distance from the event to the disturbance-affected zone increases; (**c**) the mechanism term increases with the shear/compression energy ratio, and the gray background bands indicate low-ratio, mixed, and shear-dominated ranges; (**d**) statistical-evolution term, where red shaded intervals indicate activation windows; (**e**) suppressive effect of the event-quality term, with the color background indicating quality level; and (**f**) final candidate structural activation index and its threshold-based classification results.

Figure 8a–f further show how each evidence term controls the final recognition result. Figure 8a indicates that the spatial clustering term highlights densely distributed event regions, preventing isolated noise events from being interpreted as structural activation. Figure 8b shows that the disturbance-coupling term gradually decreases as an event moves away from the face-disturbance affected zone. Figure 8c shows that the mechanism term can introduce the shear/compression energy ratio into the recognition process, so that events with stronger shear or mixed-failure characteristics receive higher contributions. Figure 8d characterizes event-activity evolution at the statistical-window scale; when the event count, energy index, and apparent volume increase while the b-value decreases, the statistical-evolution term increases accordingly. Meanwhile, the event-quality term in Figure 8e suppresses events with large picking uncertainty and high localization residuals, ensuring the reliability of the final index. Finally, Figure 8f aggregates the evidence terms into $I_{sa,k}$ and uses two threshold levels to classify low-index events and high-confidence candidate activation events.

**Figure 9:**
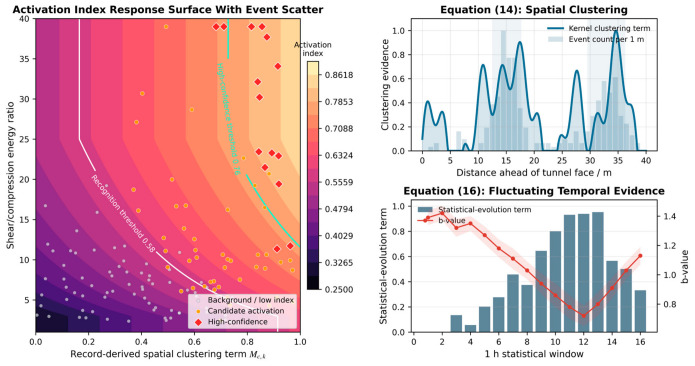
Effect of waveform- and event-catalog-evidence-driven structural activation recognition.

Figure 9 summarizes how the evidence terms are mapped to the candidate activation index; high-index events require spatial concentration, mechanism evidence, temporal evolution, and acceptable event quality to coincide.

### 2.8. Validation Scheme and Comparison Methods

To ensure that each module has a quantifiable validation basis, this study constructs three manually reviewed datasets. The first is a propagating-event ground-truth set containing 286 manually reviewed and confirmed propagating events, used to evaluate detection performance. The second is a refined first-arrival set containing 136 high-confidence events and 1248 station-level arrival records, used to evaluate first-arrival picking errors and localization residuals. The third is a structural activation review set containing 96 events with spatial clustering, mechanism anomalies, temporal clustering, and posterior geological correspondence, used to examine the consistency between recognition results and engineering interpretation.

The reference datasets were established from manual review of the continuous records and candidate windows, rather than from the final SCADL output alone. For the propagating-event ground-truth set, reviewed windows were accepted as positive events only when they showed a short-duration impulsive response on multiple adjacent stations, an interpretable across-station arrival sequence, and no dominant single-trace mechanical spike. Windows dominated by long mechanical oscillation, isolated sensor impulses, or unclear station-to-station propagation were treated as non-propagating or ambiguous and were not used as positive references. For the first-arrival set, only events with clear onsets on several stations were retained, and station-level reference arrivals were checked against the three-component waveform, synthesized amplitude, and interstation arrival order. For the structural-activation review set, event locations, temporal clustering, mechanism evidence, event quality, and posterior correspondence with the exposed F04 and F02 structural planes were jointly reviewed.

The three reference datasets should be regarded as expert-curated author labels rather than externally blinded ground truth. No independent second-rater or inter-rater reliability statistic was available for this revision; therefore, subjective decisions could affect the boundary between weak propagating events, ambiguous windows, and TBM mechanical disturbances. To reduce this subjectivity, the revised labeling rules require agreement among multi-station moveout, three-component waveform shape, short event duration, stable onset order, and the absence of dominant single-station spikes; nevertheless, cross-rater labeling and additional monitoring sessions are required before claiming site-independent robustness.

Performance evaluation was then conducted by matching each algorithm output to the corresponding manual reference set. For event detection, a candidate window was counted as a true positive when it matched a manually confirmed propagating event after one-to-one temporal matching and propagation verification; an unmatched algorithm candidate was counted as a false positive, and a manually confirmed propagating event without a matched algorithm candidate was counted as a false negative. Precision, recall, and F1-score were calculated from these event-level counts, and the false-alarm rate was calculated as the number of false positives divided by the 16 h observation duration. For first-arrival picking, the absolute time difference between the algorithm pick and the station-level manual reference pick was used. Localization performance was evaluated with arrival-time residuals, cluster thickness after relative correction, and station-removal drift. Structural-activation recognition was evaluated by comparing high-confidence activation candidates with the manually reviewed activation set and the post-excavation geological sketch.

Four comparison workflows are configured: fixed-threshold triggering, single-station STA/LTA triggering, STA/LTA triggering followed by statistical abrupt-change picking, and the continuous-evidence convergence workflow proposed in this study. To verify the necessity of each step, this study further constructs ablation versions, including removal of coherence screening, removal of polarization screening, removal of the advance velocity model, and removal of relative correction of similar event pairs. The detection part reports the number of candidate windows, number of propagating events, F1-score, and false alarms per hour. The picking part reports the median absolute error and the 95th-percentile error. The localization part reports the localization residual, cluster thickness after relative correction, and drift after station removal. The recognition part reports the number of high-confidence activation events, the consistency rate with manual review, and the ahead-of-face clustering characteristics of high-risk time windows.

## 3. Results

### 3.1. Propagating Event Detection in 16 h Continuous Records

**Figure 10:**
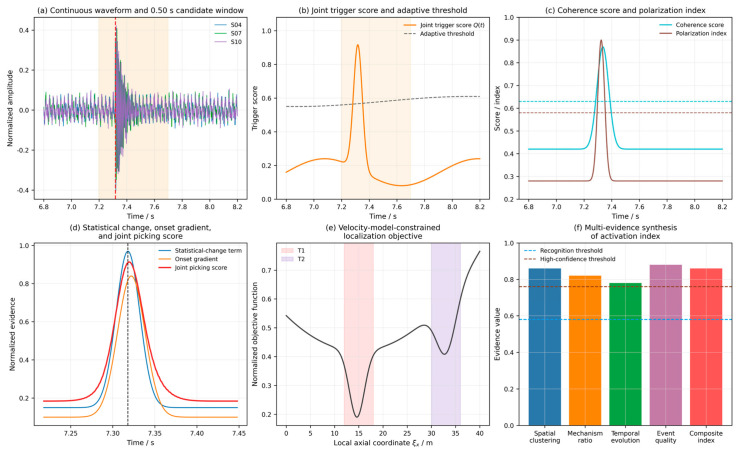
Typical candidate event window and stepwise numerical transformation in the proposed workflow. The tan background denotes the 0.50 s candidate window, dashed horizontal lines denote decision thresholds, and vertical dashed lines denote reference/pick times used in the stepwise transformation.

Figure 10 shows a typical candidate event window and the stepwise convergence from raw waveform to candidate activation criteria. Before the candidate event arrives, the three-component synthesized amplitude remains overall at a low-amplitude background level, and the joint trigger score fluctuates around the adaptive threshold. After the propagating event enters the array, at least four adjacent stations show synchronous uplift, and the trigger score is significantly higher than the threshold within the 0.50 s candidate window. At the same time, the coherence score and polarization index increase synchronously, and the first-arrival picking score forms a clear peak near the arrival time. This indicates that Equations (1)–(7) can progressively compress amplitude anomalies, across-station propagation consistency, and three-component polarization concentration into the same physical event window.

In the 16 h continuous record, the proposed workflow obtains 314 candidate windows and 263 confirmed propagating events. With respect to the 286-event propagating-event ground-truth set, these results correspond to 263 true positives, 51 false positives, and 23 false negatives after one-to-one matching. Therefore, the precision is 263/314 = 0.84, the recall is 263/286 = 0.92, and the F1-score is 0.88. The false-alarm rate is calculated as 51/16 h = 3.2 events/h, which is markedly lower than those of the fixed-threshold and single-station STA/LTA workflows. Because structural activation recognition must be built on an interpretable propagating-event catalog, the accuracy of front-end detection directly determines the reliability of subsequent picking, localization, and risk discrimination. The complete detection comparison is summarized in Table 2.

These detection metrics should therefore be interpreted as within-case performance under the adopted site-calibrated thresholds and author-curated reference labels. The present data do not allow for the separation of algorithmic robustness from possible tuning to this monitoring section; independent sessions, blinded multi-rater catalogs, and threshold-perturbation reruns are needed before transferability or robustness can be claimed.

### 3.2. First-Arrival Picking and Relative Correction

Table 3 shows that first-arrival picking quality forms a clear error-transfer constraint in subsequent localization. After the joint picking score and final pick rule in Equations (6) and (7) are applied, the median absolute first-arrival picking error is 2.4 ms and the 95th-percentile error is 4.5 ms. The advance layered velocity model in Equation (8), together with the fixed within-record timing correction, constrains the corrected localization residual to 2.9 ms. After relative correction of similar event pairs using Equation (9), the event-cluster thickness converges to 0.82 m, and the mean drift under station-removal testing is 0.49 m.

These indicators also provide a practical uncertainty and resolution check for the short linear array. When the median picking error decreases from 7.6 ms in the single-station STA/LTA workflow to 2.4 ms in the proposed workflow, the corrected localization residual decreases from 6.5 ms to 2.9 ms, the cluster thickness decreases from 1.92 m to 0.82 m, and the mean station-removal drift decreases from 1.38 m to 0.49 m. The residual and drift values therefore represent internal consistency after the adopted within-record correction and station-removal test, rather than an unconstrained absolute three-dimensional error ellipsoid or an independent validation of the velocity model.

The first-arrival picking and localization results reflect the main quality-transfer relationship in the proposed workflow. First, the joint picking score and final pick rule in Equations (6) and (7) reduce the median absolute first-arrival picking error to 2.4 ms, with a 95th-percentile error of 4.5 ms. Second, advance velocity-model-constrained localization based on Equation (8) controls the arrival-time residual to 2.9 ms. Finally, relative correction of similar event pairs based on Equation (9) further compresses event-cluster thickness to 0.82 m, and the mean drift after station removal is only 0.49 m.

Therefore, the proposed workflow does not simply pursue the minimum residual for a single event. Instead, it simultaneously examines event-pair relative geometry, station consistency, and spatial-clustering stability. The improvements in the indicators in Table 3 come from a complete constraint chain: front-end triggering first removes non-propagating noise; joint picking provides stable arrivals; the velocity model provides a travel-time kernel consistent with the stratigraphic structure; and similar-event-pair correction further removes local systematic bias.

**Figure 11:**
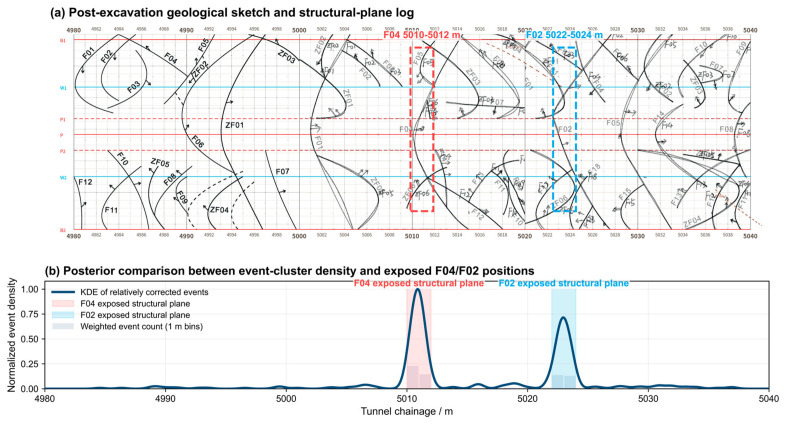
Posterior validation of velocity-model-constrained event clusters using the post-excavation geological sketch. (**a**) Simplified and target-emphasized sketch of exposed structural planes from 4980 to 5040 m; non-target structural traces are muted, while F04 (5010–5012 m) and F02 (5022–5024 m) are highlighted as the two primary validation targets in this study; and (**b**) correspondence between event density after relative correction and the exposed positions of F04 and F02.

Figure 11 compares the event clusters constrained by the velocity model with the post-excavation geological sketch to examine whether the localization results correspond to the actually exposed structural planes. It should be emphasized that the geological sketch was obtained after the tunnel face was excavated and logged; it did not participate in the detection, picking, localization, or recognition calculations in Section 2. The correspondence between the event clusters and the F04 (5010–5012 m) and F02 (5022–5024 m) structural planes is therefore an independent posterior validation.

### 3.3. Recognition of Disturbance-Induced Structural Activation Events

**Figure 12:**
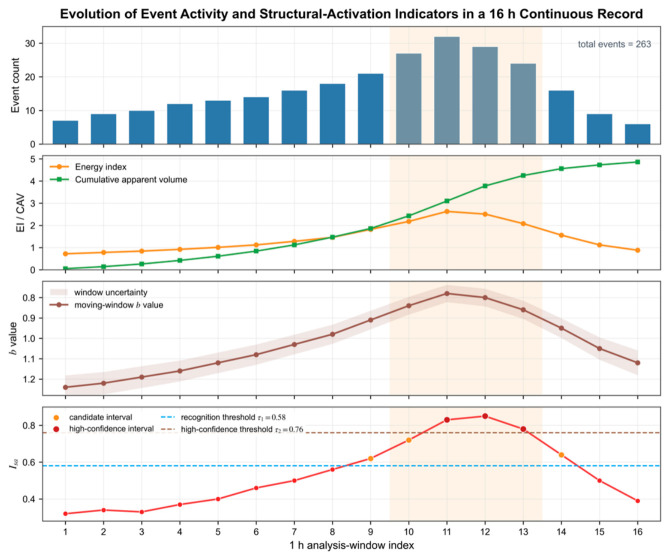
Evolution of event count, energy index, cumulative apparent volume, b-value, and structural activation index in the 16 h continuous record. The yellow background marks high-risk time windows. In these windows, the event count, energy indicators, and structural activation index increase synchronously, while the b-value decreases.

After the propagating-event catalog and velocity-model-constrained positions become stable, Equations (14)–(18) further synthesize spatial clustering, mechanism characteristics, and temporal evolution of individual events into candidate structural activation criteria. Figure 12 shows that the 16 h analysis windows cover the entire process from a low-disturbance background, through gradual event clustering, to decay of the high-risk response. When advancing from the 10th to 13th windows, the event count, relative energy index, and cumulative apparent volume increase synchronously, the window magnitude-frequency slope indicator continues to decrease, and the structural activation index enters the high-value interval. This combined change indicates that temporal statistical indicators and single-event discrimination results have a consistent risk implication.

Among all 263 valid propagating events, 86 were identified as high-confidence candidate structural activation events. In the posterior geological validation, 49 of these events were spatially associated with the F04 structural plane and 37 with the F02 structural plane. Table 4 lists representative events from the two high-confidence clusters, together with one suspected activation event and one normal surrounding-rock response, to illustrate how the event-level indicators support the final classification. High-confidence events generally show spatial clustering, elevated shear-to-compression energy ratio, increased time-window risk indicators, and high activation-index values, whereas normal surrounding-rock responses far from the exposed structural planes do not satisfy these joint criteria.

### 3.4. Sensitivity and Ablation Analysis

**Figure 13:**
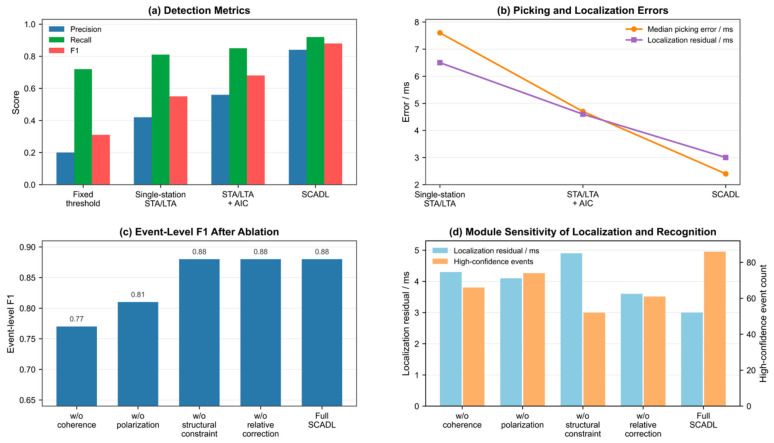
Graphical validation results for comparison workflows and ablation tests. The figure shows the detection indicators, picking and localization errors, event-level F1-score after ablation, and changes in localization residuals and numbers of high-confidence events. (**a**) Detection metrics; (**b**) picking and localization errors; (**c**) event-level F1-score after ablation; and (**d**) module sensitivity of localization and recognition.

Figure 13 and Table 5 evaluate result stability from two perspectives: overall comparison and stepwise module ablation. The overall comparison shows that the proposed workflow outperforms the comparison workflows in event detection, first-arrival picking error, and localization residual, indicating that continuous quality transfer is formed among front-end propagating-event screening, station-level arrival constraints, and velocity-model-constrained localization. However, module ablation is not equivalent to empirical-threshold sensitivity analysis.

To make the threshold issue explicit, the empirical constants are organized into four perturbation groups: trigger-generation parameters (K, α, and κ), screening and picking thresholds ($C_{th}$, $P_{th}$, cc, $\beta_{1}$, and $\beta_{2}$), activation-classification thresholds ($\tau_{1}$ and $\tau_{2}$), and activation-index weights ($\omega_{1}-\omega_{4}$). In the recommended sensitivity design, each main threshold or weight is perturbed by −20%, −10%, +10%, and +20% while the manually reviewed reference labels are kept fixed.

For each perturbation, the reported metrics should include the number of candidate windows, the number of confirmed propagating events, precision, recall, $F_{1}$-score, false alarms per hour, median picking error, localization residual, cluster thickness, station-removal drift, the number of high-confidence activation events, and the F04/F02 cluster-assignment change. This metric set links threshold variation to the four main outcomes of the workflow: event detection, arrival-time picking, localization stability, and candidate structural-activation recognition.

Because the available revision results are derived from the final catalog-level analysis and module-ablation tests, the parameter discussion is framed as a sensitivity-analysis protocol and a calibration-boundary statement rather than as an additional numerical rerun. The constants listed in Table 1 should therefore be interpreted as case-calibrated values for this monitoring section, sensor spacing, and disturbance environment. When the workflow is applied to a different lithology, array geometry, or mechanical-noise condition, these parameters should be recalibrated using local background-noise windows, representative seed events, and the same manually reviewed reference labels.

The same parameter grouping provides a practical calibration rule when the workflow is applied to another monitoring condition. The numerical coefficients in Table 1 should not be treated as universal constants. Instead, they are site- and array-specific settings that should be recalibrated using local background-noise windows, expected wave velocity and attenuation, sensor coupling, and a small manually reviewed seed set. In this calibration, K, α, and κ control candidate generation against the background-noise envelope; $S_{min}$, $P_{min}$, and ${cc}_{min}$ control coherence-polarization screening; $\beta_{1}$ and $\beta_{2}$ control arrival-picking fusion; and $\tau_{1}$, $\tau_{2}$, and $\omega_{1}$–$\omega_{4}$ control activation-index classification. Thus, what is intended to be transferred is the constrained processing sequence, whereas the absolute parameter values should be re-estimated for each monitoring section.

The ablation results further show that coherence screening and the advance velocity model are the two key physical constraints affecting the results. After coherence screening is removed, false alarms caused by the mechanical background increase, and the overall event-detection score decreases from 0.88 to 0.77. After the advance velocity model is removed, the detection indicator remains unchanged, but the localization residual increases to 4.9 ms and the number of high-confidence candidate structural activation events decreases to 52. In contrast, removing polarization screening mainly weakens the ability to distinguish weak events from mechanical noise, whereas removing relative correction of similar event pairs mainly increases event-zone thickness and spatial dispersion.

For velocity-model sensitivity, the ablation case without the advance layered velocity model can be regarded as a limiting alternative in which the travel-time kernel degenerates to a uniform velocity constraint. Under this alternative, the localization residual increases from 2.9 ms to 4.9 ms and the number of high-confidence candidate activation events decreases from 86 to 52. This result shows that the layered velocity prior materially affects cluster convergence. For picking-uncertainty sensitivity, the comparison workflows in Table 3 provide controlled perturbations of arrival quality: larger picking errors consistently lead to larger residuals, thicker clusters, and greater station-removal drift. Therefore, the conclusions are based on cluster-scale convergence that remains stable only when arrival picking, the velocity model, and relative correction are jointly constrained.

Taken together, the advantage of the proposed method does not depend on any single threshold. It comes from the role-specific convergence of multi-source evidence throughout the workflow: the detection stage produces a credible propagating-event catalog; the picking stage provides stable arrivals; the localization stage forms velocity-model-constrained event clusters without using the post-excavation geological sketch; and the recognition stage converts spatial clustering, mechanism characteristics, and temporal evolution into a candidate structural activation index. Post-excavation geological information is used only for final validation, allowing for the examination of whether the event clusters have independent engineering meaning.

## 4. Discussion

### 4.1. Relationship with Existing Methodological Routes

Most existing microseismic stability evaluations are conducted using ground arrays and are usually based on organized event catalogs to interpret phenomena such as event clustering, magnitude-frequency slope, energy release, and localization results. Multi-parameter early-warning studies further emphasize the roles of event count, energy index, cumulative apparent volume, and mechanism information in indicating risk state. On the basis of these indicators, this study fills three key gaps: detection of propagating events in continuous records, velocity-model-constrained localization of event clusters, and single-event-level candidate structural activation recognition. Thus, an indicator system that was originally more oriented toward posterior statistics can be moved forward into the interpretation process of continuous three-component near-field sensing data.

From the detection perspective, this study first addresses the difficulty of robustly discovering true propagating events under a strong mechanical background. Only when the candidate catalog has across-station propagation consistency can subsequent event clustering, temporal evolution, and multi-evidence recognition have a verifiable physical basis. From the localization perspective, this study does not introduce the post-excavation geological sketch as a constraint on absolute position during the method stage. Instead, it forms event clusters using station arrivals, the advance layered velocity model, fixed within-record timing correction, and relative time-difference relationships of similar event pairs. This design gives an internally consistent corrected location catalog, but the corrected residual should not be interpreted as independent proof of absolute velocity-model accuracy without an uncorrected-residual comparison or an independent calibration/evaluation split. From the recognition perspective, this study further implements window-level risk clues at the single-event level, corresponding to structural activation characteristics of individual events.

### 4.2. Compatibility with Current Data Conditions

The proposed method is better matched to the current in-tunnel observation system. First, although the linear near-field array composed of 12 three-component stations has a limited aperture, it provides clear constraints on interstation propagation order and local polarization characteristics. Therefore, multi-station consistency triggering, coherence screening, and relative correction of similar event pairs are more suitable for the current data conditions than end-to-end classifiers that rely on large labeled samples. Second, advance geological prospecting or prediction can provide a forward layered velocity model, giving travel-time calculation a propagation prior independent of the post-excavation geological sketch. Third, the real station coordinates and interstation arrival-time differences in the raw records transform velocity-model-constrained localization from a conceptual scheme into an executable workflow and provide spatial objects for subsequent geological validation.

More importantly, this data-processing workflow can be used when the sample size is limited. Continuous records provide temporal-evolution evidence, multi-station deployment provides propagation-consistency evidence, three-component records provide polarization constraints, and near-field relative localization provides spatial-clustering evidence for events.

### 4.3. Physical Mechanism of Excavation-Induced Structural Activation

The high-confidence event clusters identified in this study should be understood as a stress-perturbation response of pre-existing weak structural planes rather than as newly generated macroscopic fracture surfaces. During TBM advance, face unloading, cutterhead-induced disturbance, and redistribution of the near-face stress field can change the normal and shear tractions acting on discontinuities. Where a structural plane is already close to a critical slip or opening condition, a small reduction in normal confinement or a local increase in shear traction may trigger asperity failure, local slip, dilation, or mixed tensile-shear cracking. These processes can release strain energy and generate weak short-duration near-field microseismic responses. Recent analysis of mining-induced fault coseismic slip further shows that fault or structural-plane slip can redistribute strain energy around the activated zone, providing a useful physical analogue for interpreting excavation-disturbance-related event clusters [33].

This mechanism is consistent with the observations in the present case. The two main clusters are not randomly distributed in the search volume; after velocity-model-constrained localization and relative correction, they correspond to the post-excavation F04 and F02 structural planes. The activation index also requires the simultaneous convergence of spatial clustering, face-disturbance coupling, shear/compression mechanism evidence, temporal evolution, and event quality. Therefore, the term candidate structural activation is used here as an engineering interpretation supported by multi-evidence convergence and posterior geological validation. It does not imply that every retained propagating event is a directly observed macroscopic slip event; rather, the interpretation is that excavation-induced stress perturbation promoted local deformation or frictional instability on pre-existing structural planes, producing clustered weak microseismic responses.

### 4.4. Method Boundaries and Engineering Significance

Although the proposed method forms a complete loop from continuous records to geological validation under the current data conditions, its applicability boundaries should still be made explicit. First, a linear short-aperture array is inherently weaker in lateral and vertical resolution than a circular or three-dimensional network. Therefore, the results in this study are more suitable for interpreting event clusters and relative migration under velocity-model constraints and should not be understood as unconstrained high-precision three-dimensional absolute localization. Second, the layered velocity model obtained from advance geological prospecting or prediction still contains scale effects and interpretation errors; localization results should therefore be used preferentially at the cluster scale and in terms of relative spatial relationships. Third, although candidate structural activation recognition integrates spatial clustering, mechanism characteristics, statistical evolution, and event quality, indicators such as the shear/compression energy ratio, cumulative apparent volume, and magnitude-frequency slope are still affected by sample size and statistical-window length. Under extremely short records or very few events, case replay and manual review should be combined for judgment.

The influence of velocity-model geometry should therefore be considered explicitly when interpreting the locations. The advance layered model used in this study simplifies the ahead-of-face medium into several velocity zones, whereas real structural boundaries may have finite thickness, variable dip, curvature, local branching, and velocity contrasts that are resolved only at the scale of advance prospecting. Errors in boundary position, dip, or velocity contrast can produce path-dependent travel-time biases; in a short linear array, these biases are most likely to appear as residual growth, cluster elongation, or systematic shifts of event zones toward an incorrect interface or along the tunnel-axis direction. Consequently, the correspondence between the event clusters and F04/F02 should be interpreted as resolution-limited cluster-scale validation rather than exact reconstruction of the structural-plane surfaces. Future applications should perturb boundary position, dip, thickness, and velocity contrast in the a priori model, or update the model using newly exposed geology and borehole information, to bound possible location shifts.

Accordingly, the spatial correspondence with F04 and F02 should be read as a resolution-limited cluster-scale validation. The current array can resolve whether events concentrate near the expected ahead-of-face structural zones and whether those clusters migrate consistently with the velocity-model prior, but it cannot provide a unique high-resolution lateral or vertical hypocenter for every single event. This limitation is why the study reports arrival residual, cluster thickness, station-removal drift, and the velocity-model ablation result together, and why the conclusions emphasize event-zone localization and posterior geological consistency rather than absolute point-location accuracy.

Despite these boundaries, this method has direct significance for engineering practice. It shows that, in the absence of synchronous working-condition logs, large-scale manual labels, and post-excavation geological sketch priors, a verifiable near-field structural activation monitoring route can still be established as long as continuous three-component records, real station coordinates, and advance velocity information can be obtained stably. This route relies on physically constrained signal processing, velocity-model-constrained localization, relative correction of similar event pairs, and posterior geological validation. For ahead-of-face risk interpretation during TBM construction, its value lies in converting continuous vibration data into event evidence that can be localized, traced, and tested using geological information.

## 5. Conclusions

This study addresses the core problem in continuous three-component monitoring during TBM excavation: how to detect disturbance-induced structural activation events from continuous vibration records under a strong mechanical background and achieve reliable localization and recognition. A continuous-evidence convergence interpretation workflow is proposed. The workflow detects propagating events through front-end multi-station consistency triggering and coherence-polarization screening, constrains first-arrival picking through the synergy of statistical abrupt change, local gradient, and polarization, localizes event clusters through an advance layered velocity model, arrival-time residual search, and relative correction of similar event pairs, and recognizes target events using a multi-evidence-fusion candidate structural activation index.

Validation using 16 h of continuous records shows that the proposed method identifies 263 valid propagating events and achieves an event-level detection score of 0.88. The median first-arrival picking error is 2.4 ms, the corrected localization residual is 2.9 ms, and the event-cluster thickness after relative correction of similar event pairs is compressed to 0.82 m. A total of 86 high-confidence candidate structural activation events are further obtained. Validation using the post-excavation geological sketch shows that the two main event clusters correspond to the F04 (5010–5012 m) and F02 (5022–5024 m) structural planes, respectively. This indicates that continuous three-component near-field monitoring, without prior use of the post-excavation geological sketch, can still be combined with an advance velocity model to form forward structural activation monitoring results with engineering interpretability. However, the reported 2.9 ms residual is a corrected within-record residual and should be re-evaluated in future work using uncorrected-versus-corrected comparisons and an independent calibration/evaluation split.

Overall, this study should be viewed as a single-case field demonstration rather than proof of general robustness. Its main contribution is to connect continuous signal detection, velocity-model-constrained localization, and candidate structural activation recognition into a verifiable engineering interpretation chain for one 16 h TBM monitoring record. The current conclusions remain limited by the linear short-aperture array, the record duration of a single engineering project, the author-curated ground-truth labels, the absence of inter-rater reliability evaluation, and the accuracy of the velocity model. Transferability is therefore treated strictly as a future-work topic; future studies should introduce multiple engineering scenarios, independent monitoring sessions, blinded multi-rater labeling, three-dimensional array deployment, and longer continuous records to test the stability of the workflow under different geological conditions and excavation-disturbance intensities.

## Figures and Tables

**Table 1:** Key method parameters and case-adopted values.

Module	Parameter	Case-Adopted Value	Description
Preprocessing	Band-pass frequency band	20–350 Hz	Suppresses low-frequency mechanical drift and high-frequency random noise
Triggering	$n_{s}/n_{l}$	20/200	Corresponding to 20 ms and 200 ms, respectively
Triggering	K/α	4/0.55	Balances the principal-response station and common uplift of adjacent stations
Threshold	W/κ	120 s/1.8	Constructs the time-varying trigger threshold Γ(t)
Screening	$S_{min}$/mean polarization threshold	0.63/0.58	Corresponding to the coherence and polarization thresholds, respectively
Screening	Minimum number of valid stations	4	Ensures propagation consistency and geometric interpretability
Picking	Δ	3 samples	Half-width of local onset gradient
Picking	$\beta_{1}/\beta_{2}$	0.35/0.20	Weights of the polarization and gradient terms
Localization	Advance layered velocity model	v_1=4.45 km/s , v_2=4.15 km/s , v_3=3.78 km/s	Jointly determined from advance geological prospecting, advance boreholes, and rock-specimen wave-velocity tests before event localization; only a fixed station timing correction is applied afterward to compensate repeatable channel offsets
Localization	Search range	X: 0–40 m; Y: ±2.5 m; Z: ±2.0 m	Defines only the interpretation volume for velocity-model-constrained localization; does not prescribe the target structural window
Relative correction	Cross-correlation threshold	0.78	Screens highly similar event pairs
Recognition	$\tau_{1}/\tau_{2}$	0.58/0.76	Classification thresholds for the structural activation index

**Table 2:** Comparison of continuous propagating-event detection results.

Method	Candidate Windows	Confirmed Propagating Events	Precision	Recall	F1	False Alarms per Hour
Fixed threshold	1048	205	0.20	0.72	0.31	52.7
Single-station STA/LTA energy ratio	552	231	0.42	0.81	0.55	20.1
STA/LTA + statistical abrupt-change picking	438	244	0.56	0.85	0.68	12.1
Proposed workflow	314	263	0.84	0.92	0.88	3.2

**Table 3:** Comparison of first-arrival picking and localization performance.

Method	Median Picking Error/ms	95% Picking Error/ms	Corrected Localization Residual/ms	Cluster Thickness After Relative Correction/m	Drift After Station Removal/m
Single-station STA/LTA energy ratio	7.6	14.2	6.5	1.92	1.38
STA/LTA + statistical abrupt-change picking	4.7	8.8	4.6	1.28	0.91
Piecewise statistical abrupt-change criterion + polarization	3.1	5.8	3.7	1.02	0.68
Proposed continuous-evidence convergence workflow	2.4	4.5	2.9	0.82	0.49

**Table 4:** Recognition results for representative structural activation events.

Event ID	Local Coordinates $\left(\xi_{x},\xi_{y},\xi_{z}\right)$/m	Posterior Distance to Exposed Structural Plane/m	$E_{s}/E_{p}$	Window b-Value	$I_{sa}$	Review Conclusion
E-046	(10.8, 0.38, −0.24)	0.31	24.8	0.79	0.92	High-confidence structural activation (F04)
E-118	(11.4, −0.22, 0.12)	0.48	18.7	0.80	0.86	High-confidence structural activation (F04)
E-173	(22.7, 0.16, −0.07)	0.52	21.4	0.78	0.87	High-confidence structural activation (F02)
E-221	(23.6, −0.36, 0.05)	0.81	11.4	0.86	0.74	Suspected structural activation (F02)
E-249	(35.6, 0.36, 0.22)	2.31	9.6	0.95	0.63	Normal surrounding-rock response

Note: Local coordinates use the face-centered coordinate system, and $\xi_{x}$ is the distance ahead of the tunnel face.

**Table 5:** Module-ablation results and available sensitivity diagnostics.

Scheme	Event Detection F1	Median Picking Error/ms	Corrected Localization Residual/ms	High-Confidence Activation Events	Result Interpretation
Without coherence screening	0.77	3.1	4.3	66	False alarms increase and propagation-consistency evidence weakens
Without polarization screening	0.81	3.6	4.1	74	More weak events are retained, but mechanical-noise contamination increases
Without the advance velocity model	0.88	2.4	4.9	52	The travel-time kernel degenerates to a uniform velocity, increasing localization dispersion
Without relative correction of similar event pairs	0.88	2.4	3.6	61	Event-cluster thickness increases and main-zone convergence decreases
Complete continuous-evidence convergence workflow	0.88	2.4	2.9	86	Best overall performance in detection, localization, and recognition

Note: The F1-value is calculated using propagating events; the number of high-confidence activation events is determined based on $I_{sa}$ ≥ $\tau_{2}$.

## Data Availability

Data available on request from the authors.
